# Electrospun radially oriented berberine-PHBV nanofiber dressing patches for accelerating diabetic wound healing

**DOI:** 10.1093/rb/rbae063

**Published:** 2024-06-04

**Authors:** Qiuyu Wang, Sai Zhang, Jiayi Jiang, Shaojuan Chen, Seeram Ramakrishna, Wenwen Zhao, Fan Yang, Shaohua Wu

**Affiliations:** College of Textiles & Clothing, Qingdao University, Qingdao 266071, China; College of Textile and Clothing, Dezhou University, Dezhou 253023, China; College of Textiles & Clothing, Qingdao University, Qingdao 266071, China; College of Textiles & Clothing, Qingdao University, Qingdao 266071, China; State Key Laboratory of Bio-Fibers and Eco-Textiles, Qingdao University, Qingdao 266071, China; Department of Mechanical Engineering, Center for Nanotechnology & Sustainability, College of Design and Engineering, National University of Singapore, Singapore 117576, Singapore; School of Basic Medicine, Qingdao University, Qingdao 266071, China; College of Textile and Clothing, Dezhou University, Dezhou 253023, China; College of Textiles & Clothing, Qingdao University, Qingdao 266071, China; State Key Laboratory of Bio-Fibers and Eco-Textiles, Qingdao University, Qingdao 266071, China

**Keywords:** electrospinning, radial alignment, drug delivery, wound dressing, diabetic wound

## Abstract

A dressing patch made of radially oriented poly(3-hydroxybutyrate-co-3-hydroxyvalerate) (PHBV) nanofibers was successfully manufactured with a modified electrospinning strategy. The as-electrospun PHBV radially oriented nanofiber dressing patch exhibited uniform and bead-free nanofibrous morphology and innovative radially oriented arrangement, which was demonstrated to possess obviously improved mechanical property, increased surface hydrophilicity and enhanced biological properties compared to the PHBV nanofiber dressing patch control with traditionally randomly oriented pattern. Interestingly, it was found that the radially oriented pattern could induce the cell migration from the periphery to the center along the radially oriented nanofibers in a rapid manner. To further improve the biofunction of PHBV radially oriented nanofiber dressing patch, berberine (Beri, an isoquinoline alkaloid) with two different concentrations were encapsulated into PHBV nanofibers during electrospinning, which were found to present a sustained drug release behavior for nearly one month. Importantly, the addition of Beri could impart the dressing patch with excellent anti-inflammatory property by significantly inhibiting the secretion of pro-inflammatory factors of M1 macrophages, and also showed an additive influence on promoting the proliferation of human dermal fibroblasts (HDFs), as well as inhibiting the growth of *E. coli*, *S. aureus* and *C. albicans,* compared with the Beri-free dressing patch. In the animal studies, the electrospun PHBV radially oriented nanofiber dressing patch loading with high Beri content was found to obviously accelerate the healing process of diabetic mouse full-thickness skin wound with shortened healing time (100% wound closure rate after 18 days’ treatment) and improved healing quality (improved collagen deposition, enhanced re-epithelialization and neovascularization and increased hair follicles). In all, this study reported an innovative therapeutic strategy integrating the excellent physical cues of electrospun PHBV radially oriented nanofiber dressing patch with the multiple biological cues of Beri for the effective treatment of hard-to-heal diabetic wounds.

## Graphical Abstract

**Figure:**
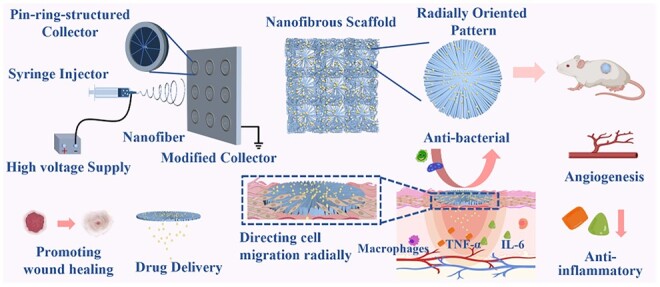

## Introduction

Diabetes is a chronic disease with a high incidence, which is always accompanied with severe complications mainly including diabetic foot ulcers, leg ulcers and other ulcers [1, 2]. According to the statistics, 25% diabetic patients are suffering the chronic injuries caused by diabetic ulcers [3, 4]. As an indispensable medical product, wound dressing has been widely employed to help the reconstruction and regeneration of damaged skin tissues [5, 6]. A relatively ideal wound dressing should be highly biocompatible, biodegradable, porous and also have strong mechanical stability, which can effectively regulate the complicated pathological mircoenvironment into the healing-promoting one, thus accelerating the wound healing in a high-quality manner [7, 8]. Unfortunately, it still lacks of appropriate dressing products to treat the diabetic wounds in clinics [9].

Although a variety of different strategies such as hydrogel and microneedle have been explored for the advanced generation of dressing patch, and each strategy has its own unique characteristics and features [10, 11], the electrospinning technique is assuredly an attractive one, which produces fibers with the diameters in the range of 50–1000 nm, which have high resemblance with the collagen nanofibrils existed in the native skin tissues in terms of morphology, structure and feature [12, 13]. In other words, electrospun nanofibers are highly biomimetic materials, which have been broadly demonstrated to provide an appropriate environment to improve the migration, proliferation, adhesion and even differentiation, as well as the secretion and remodeling of extracellular matrix (ECM) of skin-associated cells [14, 15]. In the last two decades, the initially single-fluid blending electrospinning process [16, 17] has moved forward to the coaxial [18, 19], side-by-side [20], tri-axial [21, 22], tri-fluid side-by-side [23] and their combinations [24, 25], as well as needless and surface-free electrospinning [26]. All these progresses have been inevitably based on the innovations of electrospinning spinneret [27, 28]. However, most of electrospun nanofibers are collected as the randomly oriented structure, which lack of a capacity to regulate the cell migration and control the cell orientation [29, 30]. The existing study has fabricated uniaxially aligned nanofiber mats and demonstrated their superiority compared with randomly oriented nanofiber mats [31, 32].

As reported, more than 100 different polymers have already been electrospun into nanofibers, demonstrating the excellent feasibility of electrospinning technique [33, 34]. As a renewable biopolymer, poly(3-hydroxybutyrate-co-3-hydroxyvalerate) (PHBV) has caught widespread interests for biomedical application [35, 36]. PHBV that belongs to the polyhydroxyalkanoate (PHA) family possesses excellent electrospinnability, biocompatibility and biodegradability [37]. PHBV is constructed with hydroxy-butyrate (HB) units and hydroxyvalerate (HV) units, and the HV units commonly account for 0–24%. The previous study indicated that the biodegradability of PHBV containing 3% of HV reached to ∼80% after 65 days of incubation under composting conditions on a laboratory scale [38]. PHBV has been demonstrated to possess similar mechanical properties with the polyolefins, thus serving as a promising candidate to take place of nondegradable polyolefins-based products [39, 40]. It should be noticed that one of the most important degradation product of PHBV-based scaffolds is found to be D-3-hydroxybutyric acid that is an essential ingredient of native human blood [41, 42].

The existing studies have indicated that the electrospun nanofiber-constructed dressings alone lack of enough biofunctional properties, and the healing outcomes of skin wound, especially for those chronic wounds like diabetic wound, are far from satisfactory [43, 44]. Most recently, introducing drugs, growth factors and other bioactive reagents into electrospun nanofibers seems to offer a promising route to address these issues, because electrospun nanofibers are also recognized as suitable carries for the local delivery of one bioactive ingredient in a relatively long-term manner [45, 46]. Berberine (Beri, 5,6-dihydro-9,10-dimethoxy-benzo[g]-1,3-benzodioxolo[5,6-á] quino-lizinium) is an isoquinoline alkaloid material, which is one of the most important ingredients in the traditional Chinese herbal rhizoma coptidis [47]. Beri has been demonstrated to possess anti-diabetes, antibacterial, anti-inflammatory, anti-tumor performances in the previous studies [48, 49], which seems to be a great candidate for the diabetic wound treatment [50, 51].

In this study, we aim to design and develop an innovative dressing patch constructed with PHBV radially oriented nanofibers by using our modified electrospinning strategy, which were expected to offer more appropriate physical cues to induce the migration of cells from the periphery to the center and accelerate the wound closure and healing in a faster manner, compared with the traditional electrospinning-based dressing patches made with PHBV randomly oriented nanofibers. Importantly, different concentrations of bioactive Beri were encapsulated into PHBV nanofibers during electrospinning, which were expected to impart the PHBV radially oriented nanofiber dressing patches with predetermined biological cues. In other words, we hypothesized that the Beri-loaded PHBV radially oriented nanofiber dressing patches that had ideal morphology originated from electrospun nanofibrous microstructure and radially aligned macrostructure as well as the predetermined multi-biofunctions originated from as-loaded Beri could speed up the healing process and increase the healing quality of hard-to-heal diabetic wounds.

## Material and methods

### Fabrication of PHBV randomly oriented nanofiber patch and radially oriented nanofiber patches loading without or with Beri

**Figure 1.**
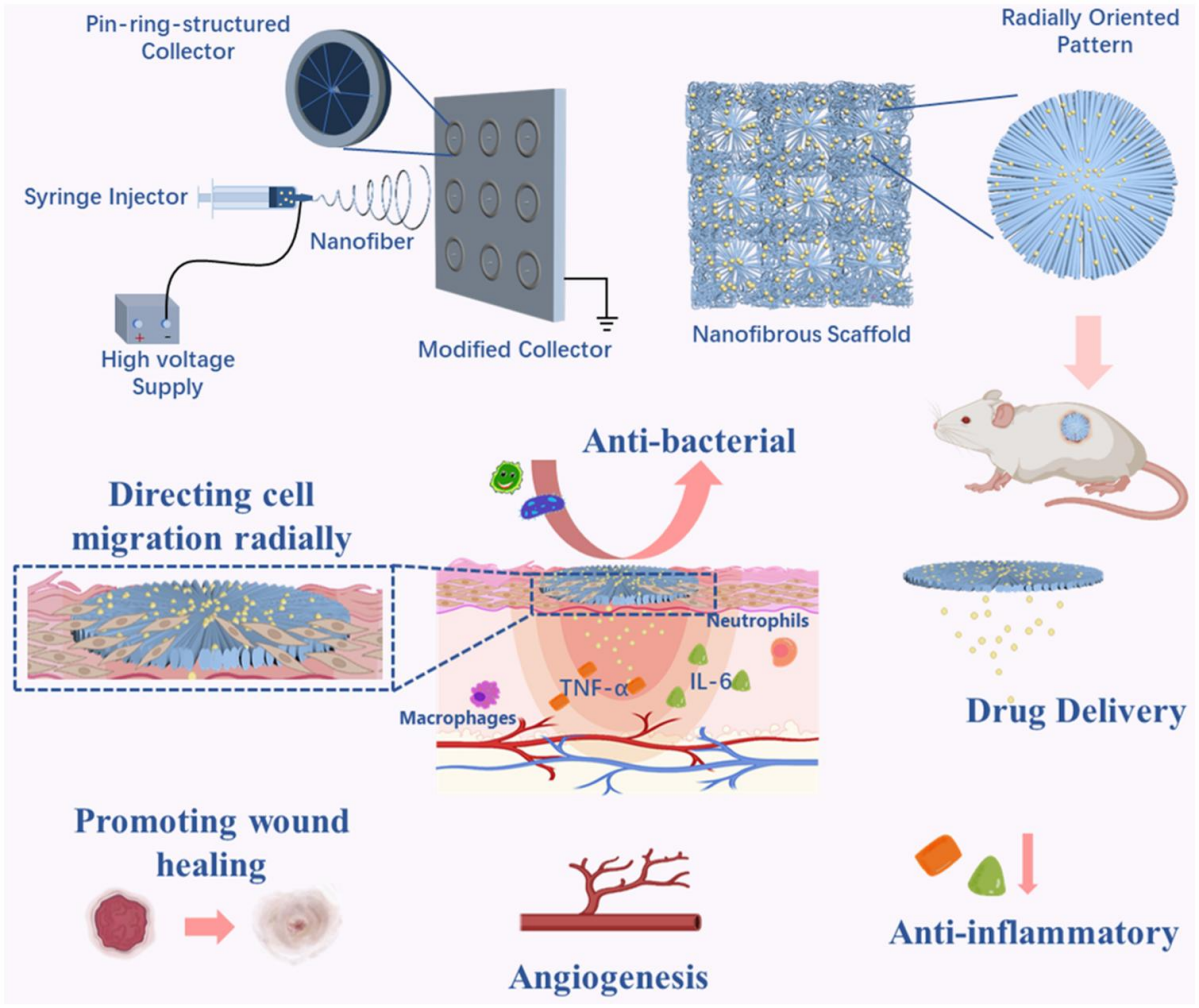
Schematic of the preparation process of Beri-loaded PHBV radially oriented nanofiber patches and their mechanisms for promoting diabetic wound healing.

An innovative electrospinning strategy was designed and used to produce both randomly oriented and radially oriented nanofiber patterns simultaneously. The core part was a modified fiber collector. In specific, a copper pin was inserted into the center of a copper ring to create a series of miniature metal collectors with pin-ring structures, and then, these pin-ring structured mini-collectors were regularly placed onto a plain plate to generate a unique fiber-collecting device (Figure 1). Together with a commercial syringe pump (longer, China), a blunt-tip needle (18 G) coupled medical syringe (10 ml) and a commercial high voltage source (Gamma, USA), a complete electrospinning device was finally obtained. PHBV (Mw = 30 000, 3% HV, Nanjing Hesu Times New Material Technology Company, China) was dissolved into hexafluoroisopropanol (HFIP, Shanghai Aladdin Reagent, China) to obtain a homogeneous spinning solution with a fixed polymeric concentration of 12% (w/v). The as-prepared solution was further electropsun into nanofiber scaffolds using our above-mentioned electrospinning device. It should be noticed that the PHBV nanofibers collected on the pin-ring structured miniature collecttors were presented in the form of radially oriented pattern, while those collected on the other areas of plain plate was found to be in the form of randomly oriented pattern. The specific electrospinning parameters were described: 15 kV spinning voltage, 18 cm collecting distance and 0.8 ml/h propulsion speed. As for the fabrication of PHBV radially oriented nanofiber patterns loading with Beri (Nanjing Zelang Medical Technology, China), Beri with two different concentrations, i.e. 1:100 and 1:20 (relative to the weight of the PHBV), were added into the 12% (w/v) PHBV spinning solution, respectively. The electrospinning device and electrospinning parameters which were totally same with the generation of Beri-free PHBV radially oriented nanofiber patterns were adopted. All the obtained PHBV nanofiber patches were vacuum dried for 72 h.

### Material characterizations

The morphology of all the different PHBV nanofiber dressing patches without or with Beri, i.e. PHBV-Randomly, PHBV-Radially, PHBV-Radially + 1%Beri and PHBV-Radially + 5%Beri, was observed by a scanning electron microscope (Regulus 8100, Hitachi, Japan). Before the observation, all the scaffold samples were subjected with a gold spraying procedure for 120 s to improve the electrical conductivity. To determine the average fiber diameter of each sample, Image J software (NIH, USA) was used, and 100 fibers from three different images were randomly chosen.

Chemical groups of each PHBV patch sample were detected by using a Fourier-transform infrared (FTIR) spectrometer (TENSOR27, Germany). The scanning resolution was set as 4 cm^−1^ with a scanning range of 4000∼500 cm^−1^.

A tester with the Cu Kα radiation source (Rigaku Ultima IV, Tokyo, Japan) was used to analyze the X-ray diffraction curves of each PHBV patch sample. The detection was performed in the range of 5° to 40° with a rate of 10°(2θ) per minute.

A universal testing machine (INSTRON 5965, USA) was employed to evaluate the mechanical characteristics of four different patch samples. Each sample was cut into a strip of 3 cm × 1cm (length × breadth), fixed with a gripping distance of 1 cm and uniaxially stretched with a tensile rate of 1 mm/min until the failure occurred. Each sample was repeated with five times. The initial modulus, ultimate strength and strain at failure were further calculated.

The surface hydrophilicity of four different patch samples were measured by a fully automatic contact measuring instrument (XG-CAMD3, Shanghai Xuanjun Instrument, China). A droplet of deionized water (2 ml) was dropped on the surface of each sample, and the dynamic changes of water contact angle were monitored over time until the equilibrium was reached.

### Drug release test

The two different Beri-loaded PHBV nanofiber patches, i.e. PHBV-Radially + 1%Beri, and PHBV-Radially + 5%Beri, were cut into 10 mg pieces, respectively. Each sample was fully immersed in 3 ml of PBS buffer (pH 7.4) in a centrifugal tube, and incubated at 37°C. The PBS solution was sampled and refilled at predetermined time points. A microplate reader (Infinite M Nano, Tecan, Switzerland) was utilized to read the OD value of the collected PBS/Berberine solution at the wavelength of 342 nm. The cumulative drug release rate was calculated, and three different replicates were adopted for each sample.

### Antibacterial assay

*Staphylococcus aureus* (*S. aureus*, ATCC 29213), *Escherichia coli* (*E.coli*, ATCC 8379) and *Candida albicans* (*C. albicans*, ATCC 10231) were employed as model microorganisms to evaluate the bacteriostatic capacity of four different dressing patches, i.e. PHBV-Randomly, PHBV-Radially, PHBV-Radially + 1%Beri and PHBV-Radially + 5%Beri. All the operation procedures were conducted according to the GB/T 20944.2-2007 standard test (China). Briefly, each sterilized patch sample was incubated with bacterial at 37°C for 24 h. The sample-free group was employed as control group. The surviving bacteria contained liquid was harvested, diluted and further coated onto an agar petridish. After additional 24 h of incubation, an automatic colonycounting apparatus (Icount 30F, Hangzhou Xunshu Technique, China) was used to record the colonyforming units. The killing ratio of bacteria for each sample were calculated with Formula 1.

(1) $$\mathrm{Killing}\;\mathrm{ratio}\;\left(\%\right)=\frac{Nc-Np}{Nc}\times 100$$

where *Nc* and *Np* were the counting number of control group and patch-contained group, respectively.

### *In vitro* cell culture and characterization

Human dermal fibroblasts (HDFs, the cell bank of the Chinese Academy of Sciences, China) were chosen as model cells to assess the biocompatibility of the as-prepared four different dressing patches. The high glucose DMEM medium (Gibco, USA) containing 10% FBS (Gibco, USA) and 1% P/S (Gibco, USA) was utilized to culture HDFs. All the cell-related experiments were performed in a 37°C incubator supplemented with 5% CO_2_. The medium was changed every other day.

To visualize how the patch pattern and structure affect the cell migration and proliferation, HDFs with a density of 1 × 10^4^ cells/20 μl were seeded on the four different points of the edge of PHBV-Randomly patch and PHBV-Radially patch with the help of specially designed template. The cell-seeded samples were cultured for 7 days. At the predetermined time interval, the samples were harvested, and a MTT assay was conducted. In specific, each sample was transferred to a 24-well plate containing 100 μl of 5 mg/ml MTT solution and 1 ml cell culture medium per well after 1, 3 and 7 days of culture. After 4 h of further culture, the blue-violet formazan crystals were completely formed, and digital photos were taken. Five hundred  microliter of dimethyl sulfoxide (DMSO) were then added to each well to totally dissolve the formazan crystals, and 100 μl liquid was harvested and added into 96-well plate. Finally, a microplate reader (Infinite M Nano, Tecan) was used to read the OD value at the wavelength of 490 nm.

Four different dressing patches, i.e. PHBV-Randomly, PHBV-Radially, PHBV-Radially + 1%Beri and PHBV-Radially + 5%Beri, were cut into 10 mg pieces and submerged in the 1 ml cell culture medium for 3 days to prepare the leaching solutions. The HDFs were seeded into 96-well plate with a density of 1 × 10^4^ cells/well, and cultured with the as-prepared leach solution through 3 days. After 1 and 3 days of culture, a MTT assay was conducted to text the indirect cytotoxicity of different patch samples. In brief, at the predetermined time point, 10 μl of MTT solution was applied to each well. The medium/MTT solution was discarded after 4 h of culture, and 100 μl of dimethyl sulfoxide (DMSO) were then added to each well to dissolve the as-formed formazan crystals. Finally, an Infinite microplate reader (Tecan) was used to record the OD value at the wavelength of 490 nm.

The murine macrophage RAW264.7 cells were purchased from Chinese Academy of Science Cell bank (China), and cultured using the medium containing high-glucose Dulbecco’s modified Eagle’s medium (Gibco), 10% FBS and 1% P/S. The RAW264.7 cells were seeded on the sterilized PHBV-Randomly, PHBV-Radially, PHBV-Radially + 1%Beri and PHBV-Radially + 5%Beri patches with a density of 1 × 104 cells per patch, and cultured for 24 h. Then, 1 μg/ml lipopolysaccharide (LPS, Suolaibao, China)-contained fresh medium was added to take place the original medium. After another 72 h of culture, the supernatant was harvested for enzyme-linked immunosorbent assay (ELISA) for each group. The cells were seeded into the 96-well plate with the same density and treatment protocol, serving as a control group. The ELISA was strictly carried out according to the manufacturer’s protocol (Sizhengbai Biological Technology, China). Two types of classical pro-inflammatory factors, i.e. tumor necrosis factor-α (TNF-α) and interleukin-6 (IL-6) were chosen to be detected.

### *In vivo* animal studies

All the *in vivo* animal experiments were approved by the Animal Research Committee of Qingdao University (Ethical approval number: QDU-AEC-2022264). The Kunming mice (female, eight-week old, Experimental Animal Center of Medical College of Qingdao University) were injected with streptozotocin (STZ, Aladdin Reagent, China) to establish the standard type 1 diabetic animal model according to our previous reports [52, 53]. The diabetic mice were then anesthetized by an intraperitoneal injection of 4% chloral hydrate and their dorsal skin surface was shaved, and a full-thickness skin defect (φ = 10 mm) at the middle of mouse back was generated with a sterilized medical puncher. To avoid the inherent contraction of skin wound, the wound site was fixed with a black silicone ring for each mouse. After the surgery, the as-prepared different dressing patches with a diameter of 10 mm, including PHBV-Randomly, PHBV-Radially, PHBV-Radially + 1%Beri and PHBV-Radially + 5%Beri, were applied to the wound bed, respectively. All the wound dressing patches were covered on the wound bed consistently without replacement. In addition, a medical gauze was employed to cover the wound bed as a control group. The wound closure in each group was monitored by taking digital photographs at days 0, 3, 6, 9, 12, 15 and 18. The corresponding wound contraction rate (%) was further determined using the Formula 2.

(2) $$\mathrm{Wound}\;\mathrm{contraction}\;\mathrm{rate}\;(\%)=\frac{{\mathrm{Area}}_{0}-{\mathrm{Area}}_{t}}{{\mathrm{Area}}_{o}}\times 100$$

where Area_0_ was the initial wound area, and Area_*t*_ was the wound area on day *t*.

The animals in each group were sacrificed at Day 18, and the regenerated tissues on the wound bed were collected for the histological analysis, and the harvested tissues were embedded with paraffin, and cross-sectioned in a series of slides with the thickness of 5 μm. The slides were analyzed by using hematoxylin and eosin (H&E) staining and Masson’s trichrome (MT) staining according to the standard procedures. In addition, the immunofluorescent staining was also employed to visualize the CD31 protein (1:200, Boaoshen Biotechnology, China) and nuclei (DAPI, 1:1000, Shanghai Lingsheng Bio-technology, China) in each group. All the stained slices were observed with a fluorescent microscope (Nikon A1 MP, Japan). The CD31 fluorescent intensity in each group was eventually analyzed with an Image J software (NIH, USA), and the relative fluorescent strength in each group was calculated using Formula 3.

(3) $$\mathrm{Relative}\;\mathrm{fluorescent}\;\mathrm{strength}\;(\mathrm{fold}\;\mathrm{of}\;\mathrm{control})=\frac{\mathrm{FSe}}{\mathrm{FSc}}$$

where FSc and FSe were the mean fluorescent strength of CD31 in the control group and the fluorescent strength of CD31 in each experimental group, respectively.

### Statistical analysis

The quantitative experiments were all conducted at least three times for each group in this work, and the mean ± standard deviation (SD) was used to describe all quantitative results. An ANOVA with Scheffé *post hoc* test was performed for the pairwise comparisons for multiple groups, and a difference of *P* < 0.05 between two groups was statistically significant.

## Results and discussion

### Fabrication and characterization of electrospun PHBV nanofiber dressing patches

**Figure 2.**
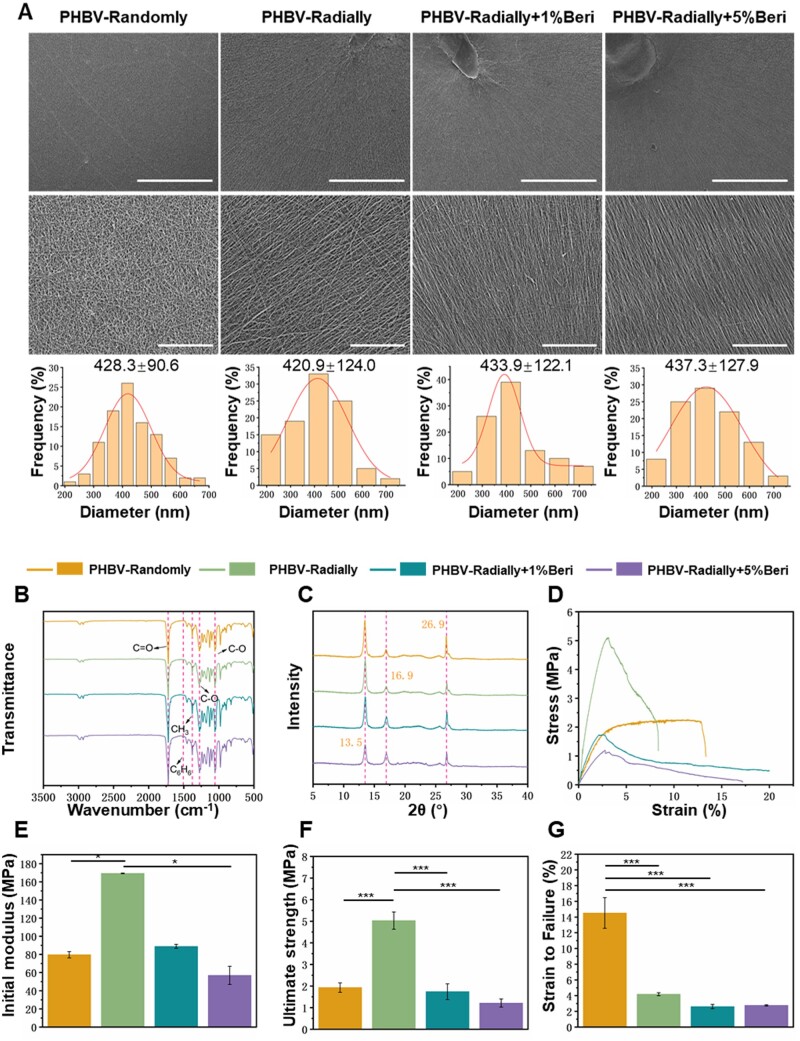
(**A**) SEM images and corresponding diameter distribution analysis of four different dressing patches, including PHBV-Randomly, PHBV-Radially, PHBV-Radially + 1%Beri and PHBV-Radially + 5%Beri. Scale bars: 500 μm for the upper panel; 100 μm for the bottom panel. (**B**) FTIR spectra and (**C**) XRD profiles of as-prepared four different PHBV nanofiber dressing patches. Mechanical properties of four different PHBV nanofiber dressing patches: (**D**) Representative tensile stress-strain curves; (**E**) Initial modulus; (**F**) Ultimate strength; (**G**) Strain at failure (*n* = 5; *P < 0.05, ***P < 0.001).

One of the most important aspect of an ideal wound dressing is its morphology and structure, which assuredly influence its physical, chemical, biological properties [38, 54, 55]. Most recently, electropspun nanofiber-based dressings are considered as promising alternatives for commercial wound dressings [56, 57]. As well known that, the typical electrospinning method employing a metal plate as fiber collector can only harvest as-generated nanofibers in the form of randomly oriented manner [58, 59]. In our present study, the plate-shaped collector was modified to collect electrospun nanofibers in both randomly oriented pattern and radially aligned design. As shown in Figure 1, a series of mini-collectors with ping-ring like structure were assembled and arranged on a plate-shaped collector. The gap formed by pin-ring structured mini-collectors were used to control the electrical field, resulting in the nanofibers deposited along with the radial direction between the copper needle and copper ring. While similar with the typical electrospinning method, the fibers deposited on the big plate collector without the mini-collectors were presented in the form of totally random pattern. Figure 2A showed the morphology and fiber diameter distribution of as-prepared PHBV randomly oriented nanofiber patches and radially oriented nanofiber patches loading without or with Beri, i.e. PHBV-Randomly, PHBV-Radially, PHBV-Radially + 1%Beri and PHBV-Radially + 5%Beri, by using our modified electrospinning device. These SEM images confirmed the feasibility and versatility of our as-developed electrospinning collector, and both randomly oriented and radially oriented fiber patches were obtained. Moreover, the addition of Beri had no obvious influences on the fiber pattern of as-obtained patches. It was also found that all the four electrospun dressing patches exhibited the smooth and bead-free fibrous morphology. The results from statistical analysis showed that the mean fiber diameter of PHBV-Randomly, PHBV-Radially, PHBV-Radially + 1%Beri and PHBV-Radially + 5%Beri was 428.3 ± 90.6 nm, 420.9 ± 124.0 nm, 433.9 ± 122.1 nm and 437.3 ± 127.9 nm, respectively, and no significant differences were detected. These fiber diameter numbers were close to the diameter number of collagen fibers in the native skin ECM, ranging from 50 to 500 nm [60, 61], indicating that all the four different PHBV dressing patches had the high skin ECM-mimicking morphology.

### FTIR and XRD characterization of electrospun PHBV nanofiber dressing patches

FTIR spectra was used to characterize the chemical groups of four different PHBV nanofiber dressing patches (Figure 2B). The characteristic peak centered at 1721 cm^−1^ was attributed to the stretching vibration of PHBV ester carbonyl (C = O), and the characteristic peak centered at 1379 cm^−1^ was attributed to the stretching vibration aliphatic group (-CH3) of PHBV [62, 63]. The symmetric stretching vibration peaks of PHBV carbon-oxygen (C-O) bond were also observed at 1278 cm^−1^ and 1055 cm^−1^. It was predictable that the above-mentioned PHBV characteristic peaks were observed for all the four different samples. In addition, the presence of berberine aromatic group was observed at 1507 cm^−1^ in the two Beri-contained samples, i.e. PHBV-Radially + 1%Beri dressing patch and PHBV-Radially + 5%Beri dressing patch [64], indicating that Beri has been successfully loaded into the PHBV nanofibers.

The XRD profiles (Figure 2C) showed that two characteristic peaks located at 13.5° and 16.9° that belonged to the α phase of PHBV, and one characteristic peak located at 26.9° corresponding to PHBV (040) crystal plane, were all detected in the four different PHBV patch samples [65]. The strength of characteristic peaks presented a decreased trend with the Beri content increasing, indicating that the addition of Beri could decrease the crystallinity of as-prepared PHBV nanofiber dressing patch to some extent.

### Mechanical properties of electrospun PHBV nanofiber dressing patches

Mechanical performances that can ensure the structural stability and movement tension tolerance of a dressing on the wound bed should also be considered [66]. The tensile mechanical properties of the PHBV-Randomly, PHBV-Radially, PHBV-Radially + 1%Beri and PHBV-Radially + 5%Beri nanofiber dressing patches were determined and presented in Figure 2D–G. Figure 2D clearly showed that the typical stress-strain curve of PHBV-Randomly dressing patch was totally different from the other three ones due to the different fibrous patterns. A linear region and an obvious yield platform was found for the PHBV-Randomly dressing patch. As control, the other three dressing patches with radially aligned patterns showed no yield platform regions, and broke in a gradually breaking manner after the linear region. Figure 2E showed that the PHBV-Radially dressing patch exhibited the highest initial modulus (169.3 ± 0.5 MPa), and the initial modulus for the PHBV-Randomly, PHBV-Radially + 1%Beri and PHBV-Radially + 5%Beri dressing patches was only 79.7 ± 3.5 MPa, 88.9 ± 2.2 MPa and 56.9 ± 10.0 MPa, respectively. Figure 2F displayed that the ultimate stress was 1.9 ± 0.2 MPa for the PHBV-Randomly dressing patch, 5.0 ± 0.4 MPa for the PHBV-Radially dressing patch, 1.7 ± 0.4 MPa for the PHBV-Radially + 1%Beri dressing patch, and 1.2 ± 0.2 MPa for the PHBV- Radially + 5%Beri dressing patch, and the ultimate stress in the PHBV-Radially dressing patch group was demonstrated to be significantly higher than the other three groups. Figure 2G indicated that the strain to failure of PHBV-Random dressing patch (14.5 ± 2.0%) was significantly larger compared with PHBV-Radially dressing patch (4.2 ± 0.2%), PHBV-Radially + 1%Beri dressing patch (2.6 ± 0.3%) and PHBV-Radially + 5%Beri dressing patch (2.8 ± 0.1%). Taken together, the mechanical characteristics of nanofibrous dressing patches were significantly influenced by the fiber arrangement and drug loading. The radially aligned fibrous arrangement could enhance the initial modulus and ultimate stress in a significantly higher manner, but the addition of Beri could obviously reduce the mechanical properties of as-obtained dressing patches.

### Surface hydrophilicity and drug release behavior of electrospun PHBV nanofiber dressing patches

**Figure 3.**
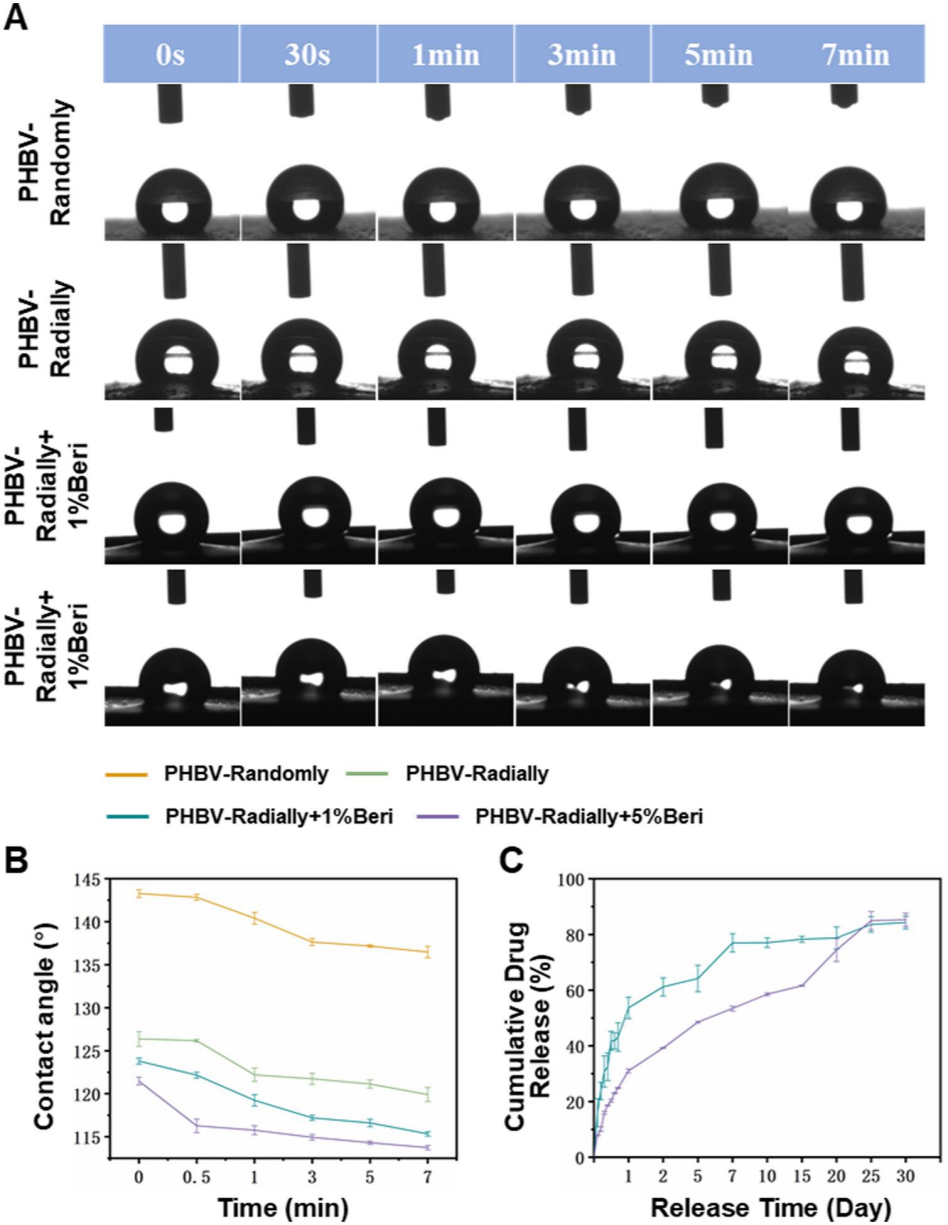
(**A**) Actual photographs and (**B**) Quantitative analysis of water contact angle of as-prepared four different PHBV dressing patches with or without Beri. (**C**) Time course of the sustained Beri release from the two different Beri-loaded PHBV nanofiber dressing patches.

The surface hydrophilicity of biomaterial patches is crucial because numerous prior studies have shown that the surface hydrophilicity of nanofiber patches significantly influences cell adhesion, proliferation and migration [67–69]. As shown in Figure 3A and B, the surface hydrophilicity of four different samples including PHBV-Randomly, PHBV-Radially, PHBV-Radially + 1%Beri and PHBV-Radially + 5%Beri was characterized by the water contact angle test. The initial water contact angle of PHBV-Randomly was much higher than PHBV-Radially, PHBV-Radially, PHBV-Radially + 1%Beri and PHBV-Radially + 5%Beri (143.3 ± 0.4° vs. 126.4 ± 0.9°, 123.8 ± 0.4° and 121.4 ± 0.5°). The water contact angle of the four samples in the equilibrium status still uphold this principle (136.5 ± 0.7° vs. 119.9 ± 0.8°, 115.3 ± 0.2° and 113.7 ± 0.3°). The results indicated that both the radially oriented arrangement of nanofibers and the loading of Beri could improve the surface hydrophilicity of dressing patches to some extent. Both material and structure of one dressing patch are responsible for its surface hydrophilicity. The radially aligned fiber structure may provide the guidance to promote the water droplet move along the fiber oriented direction to some extent. Some existing studies have indicated the wet environment is beneficial for the wound healing, and that’s the reason why the hydrogel-based wound dressings have been widely investigated in the recent years [70, 71]. Therefore, much endeavor should be conducted to further improve the surface hydrophilicity of PHBV-based wound dressing in the near future.

The cumulative drug release curve of two Beri-loaded PHBV nanofiber dressing patches were shown in Figure 3C. At Day 1, the cumulative release rate was detected to be ∼53% and ∼31% for the PHBV-Radially + 1%Beri dressing patch and PHBV-Radially + 5%Beri dressing patch, respectively, indicating an obviously initial burst release behavior. This phenomenon was totally agreed with the drug release behavior of electrospun nanofibers reported by the previous studies [45, 72]. After Day 1, the Beri release was presented in a relatively slow manner for both of the PHBV-Radially + 1%Beri dressing patch and PHBV-Radially + 5%Beri dressing patch. At Day 30, the roughly 85% cumulative release rate was found for both of the HBV-Radially + 1%Beri dressing patch and PHBV-Radially + 5%Beri dressing patch. The continuous release of Beri was expected to allow the dressing patches with a sustained antibacterial, anti-inflammatory effect. However, the release mechanism of Beri from the PHBV nanofibers is not clear now, and much more efforts will be made to explore it in the future.

### Antibacterial properties of electrospun PHBV nanofiber dressing patches

**Figure 4.**
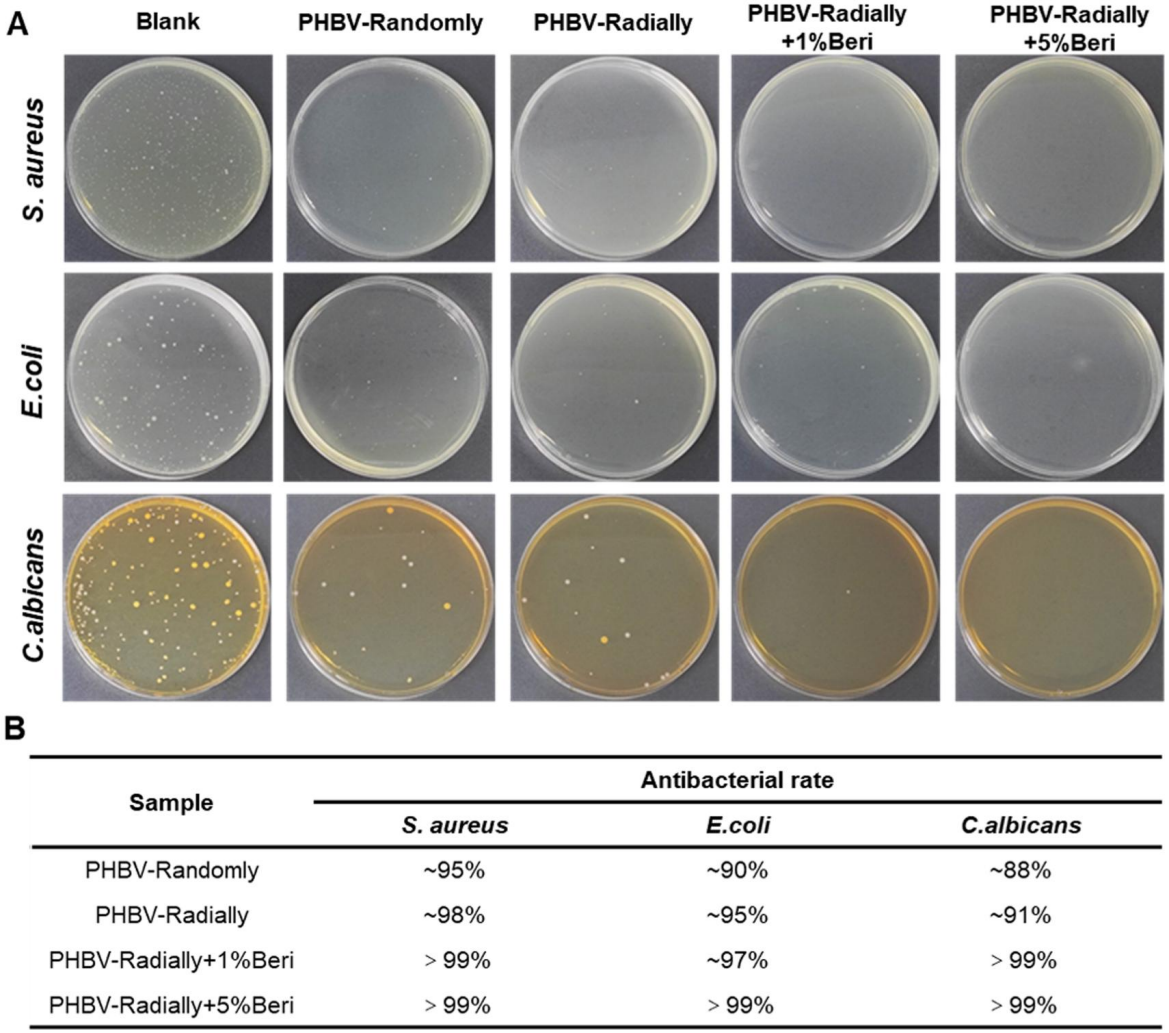
(**A**) Digital photographs and (**B**) Antibacterial rate of as-prepared four different PHBV dressing patches with or without Beri against *S.aureus*, *E.coli* and *C. albicans*.

Chronic wounds are more prone to bacterial infection due to their delayed healing process [73]. A microbial infection at the wound site can significantly slow down the healing, further cause necrosis and sepsis, and even result in death [74]. Therefore, the antibacterial properties are essential for the fabrication of dressing patches for the treatment of chronic wounds [75]. It has been reported that PHBV can destroy the cell membrane of bacteria, resulting in metabolic imbalance, thus, inhibiting bacterial growth, and Beri can disrupt the structural integrity of bacteria, block their gene expression and protein synthesis for the bacteriostatic purposes [76, 77]. *S.aureus*, *E.coli* and *C.albicans* were chosen as model bacterial to test the antibacterial properties of as-prepared four different PHBV dressing patches with or without Beri. Serving as a physical barrier between the wound and its surrounding environment, the nanofiber dressings should have strong antibacterial characteristics, and the bacterial killing capacity of four different PHBV nanofiber dressing patches for *S.aureus*, *E.coli* and *C.albicans* were shown in Figure 4A and B. The outcomes displayed that the four patch samples were ∼95%, ∼98%, >99% and >99% effective at killing *S. aureus*, respectively. The killing rate for *E. coli* was ∼90%, ∼95%, ∼97% and >99% for the PHBV-Radially, PHBV-Radially, PHBV-Radially + 1%Beri and PHBV-Radially + 5%Beri dressing patches, respectively. Moreover, the killing rate for *C. albicans* was ∼88%, ∼91%, >99% and >99% for the PHBV-Radially, PHBV-Radially, PHBV-Radially + 1%Beri and PHBV-Radially + 5%Beri dressing patches, respectively. Generally, the PHBV nanofibers exhibited strong antibacterial activity, especially for *S. aureus*, and the addition of Beri could further improve the antibacterial performances of as-prepared PHBV nanofiber dressing patches.

### *In vitro* cell characterization of electrospun PHBV nanofiber dressing patches

**Figure 5.**
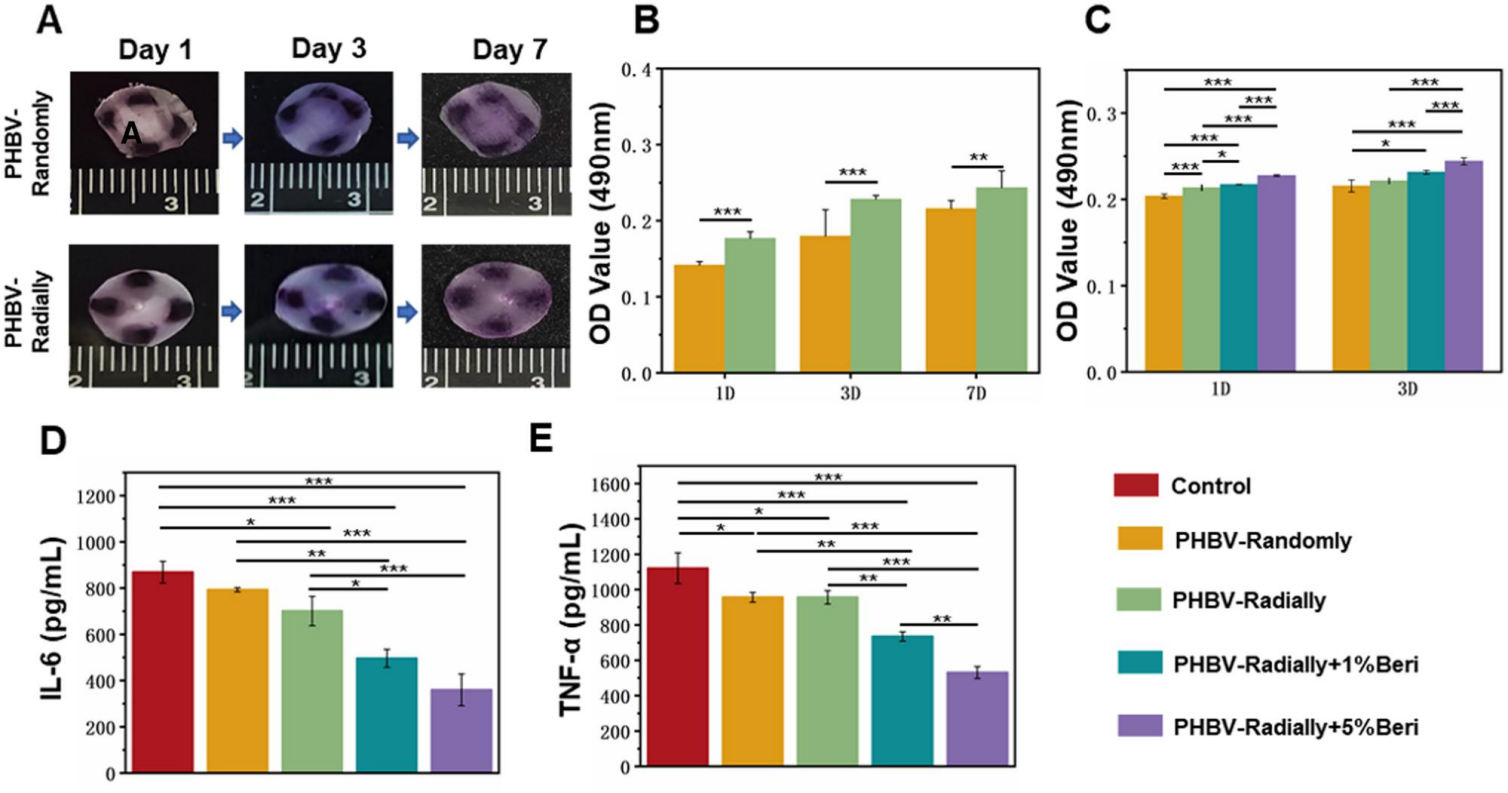
(**A**) Migration characterization and (**B**) MTT assay of HDFs seeded on the four different points of the edge of the PHBV-Randomly and PHBV-Radially nanofiber dressing patches throughout 7 days of culture (*n* = 5; **P < 0.01, ***P < 0.001). (**C**) Indirect cytotoxicity of all the four different PHBV nanofiber dressing patches with or without beri (*n* = 5; *P < 0.05, ***P < 0.001). Secretion levels of (**D**) IL-6 and (**E**) TNF-α of LPS-activated RAW264.7 cells seeded on the four different PHBV nanofiber dressing patches at Day 3 using ELISA kits (*n* = 3; *P < 0.05, **P < 0.01, ***P < 0.001).

One of the crucial factors of an excellent wound dressing is its capacity to control the cell behavior and create an outstanding micro-environment for promoting wound healing [78, 79]. HDFs have been widely demonstrated to produce and remodel the ECM of skin tissues, which can also communicate with other cell types and play a crucial role in regulating the regeneration and healing of skin wounds [80]. HDFs were first seeded on four different points of the edge of PHBV-Randomly dressing patch and PHBV-Radially dressing patch to investigate how the fiber arrangement affect the cell migration behavior. As shown in Figure 5A, the cells seeded on the PHBV-Radially patch could migrate centripetally along with the radially oriented nanofibers, while the cells cultured on the PHBV-randomly patch mainly stayed on the original position, and no obvious migration behavior was found. Importantly, some cells on the PHBV-Radially patch have already migrated into the center of the dressing patch on Day 7. The results indicated that the nanofiber alignment could definitely regulate the cell migration direction, which was consistent with previous researches [81, 82]. The MTT assay (Figure 5B) was further utilized to assess the adhesion and proliferation behavior of HADMSCs on both PHBV-Randomly and PHBV-Radially patches, and the results showed that the PHBV-Radially patch could significantly promote the cell adhesion and proliferation compared to the PHBV-Randomly patch through 7 days of culture. As well known that the reduced migration and proliferation abilities of fibroblasts on the diabetic wound sites are one of the most important factors, which hold back the wound healing [83, 84]. From this prospective, the prepared PHBV dressing patch with radially oriented fibrous pattern may be beneficial for guiding the rapid migration of the autologous cells from the wound periphery to the center, thus promoting the wound healing rate.

In our present study, Beri with different concentrations have been successfully incorporated into PHBV radially oriented fiber patches, which have been demonstrated to exhibit a sustainable release behavior (Figure 3C). MTT assay was conducted to assess HDF viability and proliferation of Beri-loaded PHBV radially oriented fiber patches with two different drug concentrations in an indirect manner (Figure 5C). The results displayed that the OD value of the PHBV-Radially + 5%Beri group were highest at days 1 and 3, and even the PHBV-Radially + 1%Beri group that possessed pretty low drug also exhibited higher OD values compared to the Beri-free groups at days 1 and 3, indicating that the addition of Beri could significantly promote the growth and proliferation of HDFs. Therefore, the prepared Beri-contained PHBV radially oriented dressing patch may be useful for accelerating the wound healing rate. Self-circulation of chronic inflammation is another important factor that makes the diabetic wounds stay at the long hard-to-heal phase [85]. IL-6 and TNF-α are two of typical pro-inflammatory factors and their elevated expression levels are the characteristics of the protracted inflammatory phase [83]. The LPS-activated RAW264.7 cells were chosen to characterize the inflammatory regulation capacity of the PHBV-Randomly, PHBV-Radially, PHBV-Radially + 1%Beri and PHBV-Radially + 5%Beri dressing patches. RAW264.7 cells were cultured in the 96-well plate, and also activated with LPS, which were utilized as control group. The ELISA results displayed that the PHBV-Radially + 5%Beri group had the lowest expression levels of both IL-6 and TNF-α, followed by the PHBV-Radially + 1%Beri group (Figure 5D and E), which indicated that the Beri-loaded PHBV radially oriented nanofiber dressing patches could significantly decrease the secretion of proinflammatory factors that were assuredly beneficial for the treatment of diabetic wounds.

### *In vivo* animal studies of electrospun PHBV nanofiber dressing patches

**Figure 6.**
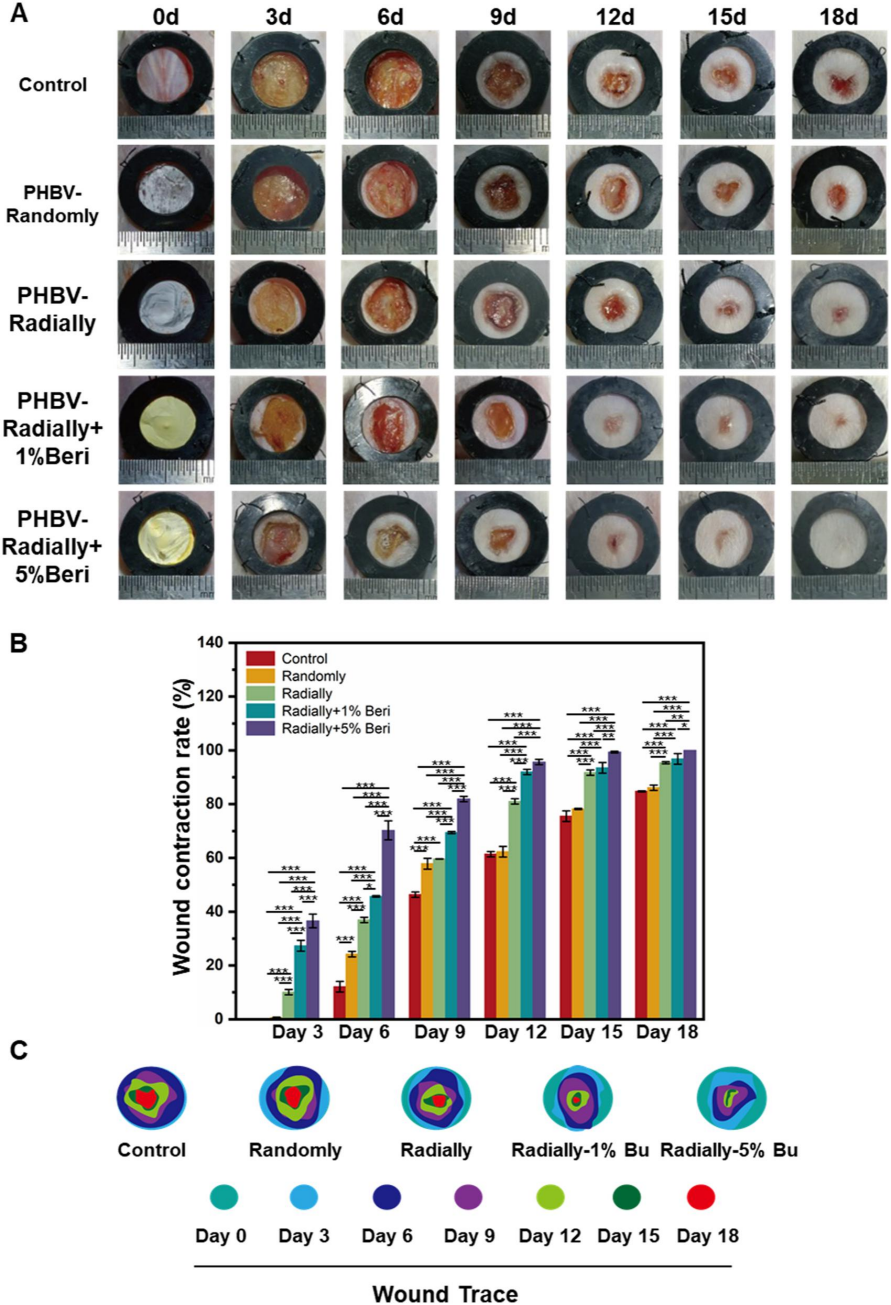
(**A**) Digital photographs of the wound sites for control group, PHBV-Randomly group, PHBV-Radially group, PHBV-Radially + 1% group and PHBV-Radially + 5% group at Days 3, 6, 9, 12, 15 and 18 after surgery. (**B**) Quantitative analysis of the wound contraction rate for the five different groups at the predetermined time points (*n* = 3; *P < 0.05, **P < 0.01,***P < 0.01). (**C**) Schematic diagram of the wound closure with the time increasing for the five different groups.

In order to investigate the therapeutic efficiency of as-prepared four different dressing patches, i.e. PHBV-Randomly, PHBV-Radially, PHBV-Radially + 1%Beri and PHBV-Radially + 5%Beri, the full-thickness skin defect using a mouse model of type 1 diabetes was established. The gross observation using digital photographs of wound site showed that the wounds in each group presented a tendency to close gradually with the treatment time increasing (Figure 6A). The PHBV-Radially + 1%Beri group was very close to complete healing at Day 18, and the PHBV-Radially + 5%Beri group exhibited a complete healing at Day 18. Quantification results of the wound healing rate showed that obviously higher healing rates were observed in the PHBV-Radially group compared with the PHBV-Radially group throughout the 18 days of treatment (Figure 6B), indicating that the radially oriented pattern could offer an effective contact guidance function to autologous cells, thus resulting in the rapid migration of autologous cells from the periphery to the wound center. In addition, the wound closure rate in the two Beri-loaded group was significantly higher (∼97% for the PHBV-Radially + 1%Beri group and ∼100% for the PHBV-Radially + 5%Beri group) than that of the control group (∼85%), the PHBV-Randomly group (∼86%) and the PHBV-Radially group (∼95%), indicating that the loading of Beri could dramatically accelerate the closure of diabetic wound. In order to clearly compare the wound closure with the time increasing for the five different groups, the schematic diagram of simulating the dynamic healing process was conducted and presented in Figure 6C.

**Figure 7.**
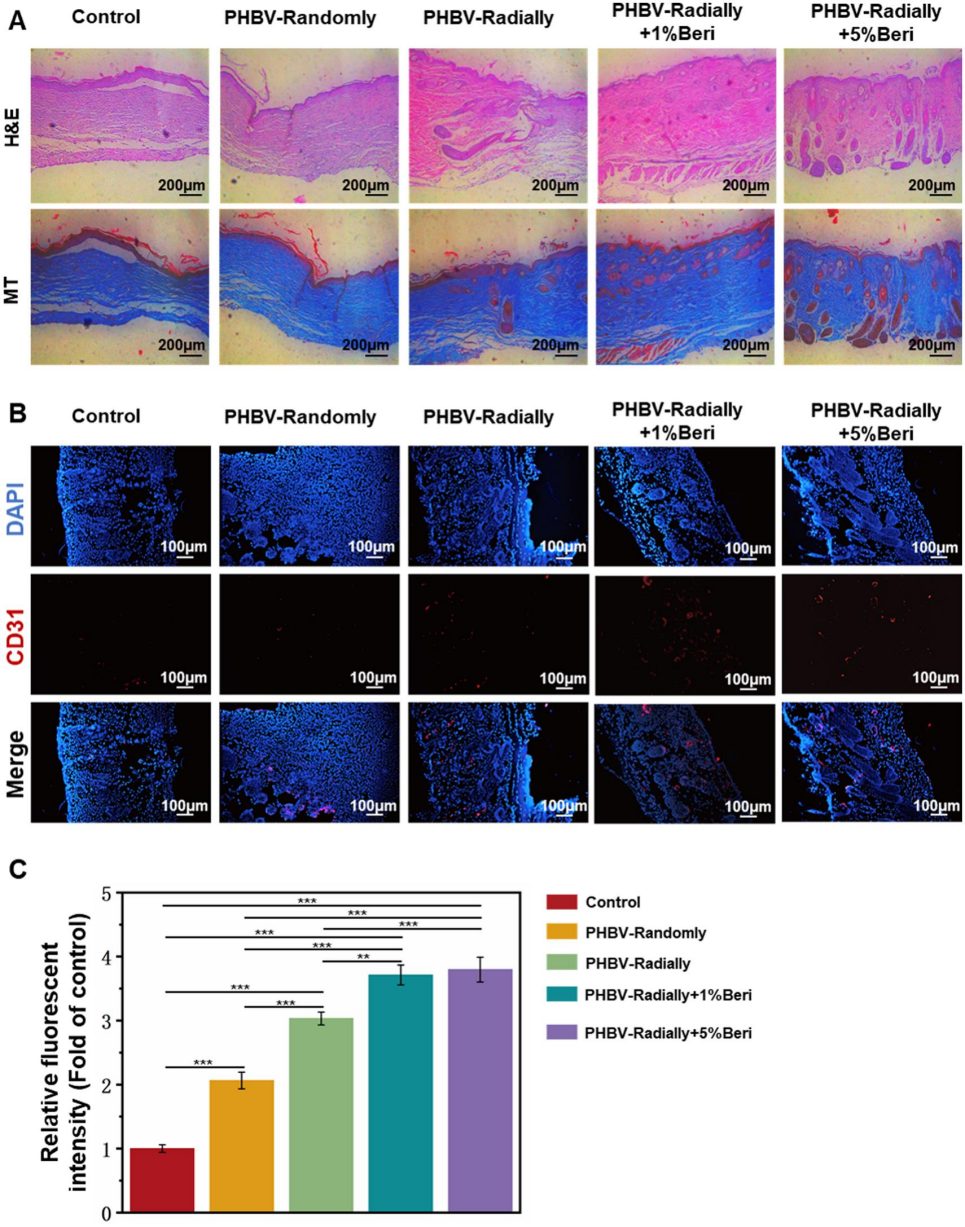
(**A**) Images of H&E and MT staining of regenerated skin tissues in the groups of control, PHBV-Randomly, PHBV-Radially, PHBV-Radially + 1%Beri and PHBV-Radially + 5%Beri at day 18 after surgery. (**B**) CD31 and DAPI fluorescent images of the regenerated skin tissue at the wound site at day 18 after surgery. (**C**) Quantitative analysis of CD31 intensity for five different groups (*n* = 3; **P < 0.01, ***P < 0.001).

At Day 18 after the treatment, the regenerated tissues on the wound site in the five different groups were harvested to conduct H&E staining and MT staining, respectively (Figure 7A). Compared with the control group and PHBV-Randomly group, the skin defect in the PHBV-Radially, PHBV-Radially + 1% Beri group and PHBV Radially + 5%Beri groups tended to be renewed, and the relatively matured neo-epidermis layers were observed in the PHBV-Radially + 1%Beri, PHBV-Radially + 5%Beri groups. A large number of collagen deposition was observed in each group, but obviously more matured collagens were detected in the PHBV Radially + 5%Beri group, which was most similar to those in the normal skin tissue. In addition, no evident regeneration of hair follicles were found in the control and PHBV-Randomly groups, but an obvious regeneration of PHBV-Randomly group was found in the groups of PHBV-Radially, PHBV-Radially + 1%Beri, PHBV-Radially + 5%Beri, respectively. Moreover, the number of regenerated hair follicles treated with the PHBV-Radially + 5%Beri dressing patch was highest among the five groups.

In the diabetic wound bed, the inferior vascularization accompanying with ischemia negatively affects the delivery of nutrients and wastes, resulting the delayed wound healing [86, 87]. To characterize the formation of new blood vessels on the wound sites, the immunofluorescent staining of CD31, a marker of vascular endothelial cells, was carried out (Figure 7B). Some more CD31 proteins in the PHBV-Radially + 1%Beri, PHBV-Radially + 5%Beri groups were detected than that in the other three groups. The semi-quantitative characterization displayed that the relative fluorescence intensity of CD31 in the PHBV-Randomly, PHBV-Radially, PHBV-Radially + 1%Beri and PHBV-Radially + 5%Beri groups was 2.1 ± 0.1 folds, 3.0 ± 0.1 folds, 3.7 ± 0.2 folds and 3.8 ± 0.2 folds compared with the control group, respectively, (Figure 7C). These results revealed that the PHBV-Radially + 5%Beri dressing patch could dramatically promoted the angiogenesis of regenerated skin tissues on the wound bed.

## Conclusion

In this study, a modified electrospinning strategy was successfully designed and developed to collect PHBV nanofibers in the form of an innovative dressing patch with a radially oriented fibrous pattern, which was demonstrated to be a more ideal dressing structure compared with the traditionally randomly oriented pattern also made from PHBV nanofibers. For instance, the radially oriented pattern could obviously improve the surface hydrophilicity and mechanical properties of dressing patch in comparison with the randomly oriented control. Importantly, compared with the randomly oriented PHBV nanofiber dressing patch, the biological properties of radially oriented PHBV nanofiber dressing patch were also dramatically enhanced by effectively guiding the migration of HDFs from the periphery to the center along the radially oriented nanofibers, and also dramatically promoting the adhesion and proliferation of HDFs. Furthermore, Beri was encapsulated into PHBV radially oriented nanofiber dressing patch during electrospinning, which was demonstrated to effectively deliver Beri in a sustained drug release manner. The Beri-loaded PHBV radially oriented nanofiber dressing patch was found to dramatically improve the antibacterial properties, anti-inflammatory activity and biological properties compared with Beri-free PHBV dressing patch control. The animal studies further demonstrated that the PHBV-Radially + 5%Beri dressing patch could largely shorten the healing period of diabetic mouse full-thickness skin wound. In specific, the wound closure rate was found to be 100% once the wound was treated with the PHBV-Radially + 5%Beri dressing patch for 18 days. Moreover, the re-epithelialization, neovascularization, collagen deposition, hair follicle regeneration of new-formed tissues were dramatically increased and enhanced in the group treated with the PHBV-Radially + 5%Beri dressing patch at Day 18, indicating the significantly improved healing quality compared with control and other groups. Based on these results, the new-type Beri-loaded PHBV radially oriented nanofiber dressing patch possessed synergistic effects that were originated with the radially oriented structure of electrospun PHBV nanofibers and the multiple biofunctions of Beri, which opens a new perspective in the effective treatment of hard-to-heal diabetic wounds. Together with the results from some existing studies, it has been demonstrated that appropriate dressing structure combined with multiple biofunctions are of significant importance for the design and development of innovative wound dressings. The involved healing mechanisms and big animal studies of our as-prepared Beri-loaded PHBV radially oriented nanofiber dressing patch will be investigated in the near future.

## Funding

This work was supported by State Key Laboratory of Bio-Fibers and Eco-Textiles of Qingdao University [TSKT202102] and Start-up Grant of Qingdao University. This work was also supported by the National Natural Science Foundation of China [22104010], Shandong Provincial Natural Science Foundation of China [ZR2021QB141] and Qingchuang Science and Technology Plan of Shandong Province [2023KJ271].

*Conflicts of interest statement*: The authors declare that they have no known competing financial interests or personal relationships that could have appeared to influence the work reported in this paper.
